# Inflated Sporopollenin Exine Capsules Obtained from Thin-Walled Pollen

**DOI:** 10.1038/srep28017

**Published:** 2016-06-15

**Authors:** Jae Hyeon Park, Jeongeun Seo, Joshua A. Jackman, Nam-Joon Cho

**Affiliations:** 1 School of Materials Science and Engineering Nanyang Technological University, 50 Nanyang Avenue 639798, Singapore; 2 Centre for Biomimetic Sensor Science, Nanyang Technological University, 50 Nanyang Drive 637553, Singapore; 3 School of Chemical and Biomedical Engineering, Nanyang Technological University, 62 Nanyang Drive 637459, Singapore

## Abstract

Sporopollenin is a physically robust and chemically resilient biopolymer that comprises the outermost layer of pollen walls and is the first line of defense against harsh environmental conditions. The unique physicochemical properties of sporopollenin increasingly motivate the extraction of sporopollenin exine capsules (SECs) from pollen walls as a renewable source of organic microcapsules for encapsulation applications. Despite the wide range of different pollen species with varying sizes and wall thicknesses, faithful extraction of pollen-mimetic SECs has been limited to thick-walled pollen capsules with rigid mechanical properties. There is an unmet need to develop methods for producing SECs from thin-walled pollen capsules which constitute a large fraction of all pollen species and have attractive materials properties such as greater aerosol dispersion. Herein, we report the first successful extraction of inflated SEC microcapsules from a thin-walled pollen species (*Zea mays*), thereby overcoming traditional challenges with mechanical stability and loss of microstructure. Morphological and compositional characterization of the SECs obtained by the newly developed extraction protocol confirms successful protein removal along with preservation of nanoscale architectural features. Looking forward, there is excellent potential to apply similar strategies across a wide range of unexplored thin-walled pollen species.

The evolutionary adaptation of pollen capsules with sophisticated wall features has been a key factor in the inhabitation and prevalence of plants^1^,^2^. Composed of a bilayer arrangement with an inner intine layer and outer exine layer^3^,^4^,^5^,^6^, the pollen wall constitutes a highly adaptable and robust structure that can endure harsh environmental conditions and chemical treatments^7^,^8^. The physical robustness of the pollen wall is attributed to the rigid intine layer composed of cellulose, hemicellulose, and lignin, whereas the exine layer composed of a biopolymer known as sporopollenin contributes by enhancing the durability of the wall with sporopollenin-specific properties^2^,^4^,^6^,^8^,^9^,^10^,^11^. The characteristic ornamentations of sporopollenin that appear on pollen surfaces as well as the size of pollen grains have diverged with the evolution of plant species^9^,^12^, but all members of the sporopollenin family have retained important physicochemical properties in order to ward off UV light-induced damage, prolonged dessication, oxidative free radicals, and microbial attack^4^,^13^,^14^,^15^.

Pollen grains are fundamentally microscale delivery vehicles containing sensitive information that needs to be protected and sustained until being received by female reproductive structures^4^. Being the first line of defense, sporopollenin is equipped with various evolutionary-driven resilient properties specifically tailored for protection and endurance to stay viable for extended lengths of time. Due to these attractive properties along with a large and renewable supply, pollen grains have gained the attention of the wider scientific community and are proving to be excellent microcapsules for encapsulation applications^3^,^16^,^17^. In order to address possible issues of biocompatibility and allergenicity, a protein-free microcapsule derivative known as a sporopollenin exine capsule (SEC) was developed through an extraction process that leaves only the hollow sporopollenin exine layer, which itself functions as a microcapsule and can be utilized for encapsulation applications^15^,^18^,^19^,^20^,^21^,^22^. As a microcapsule, SECs have demonstrated great versatility for applications such as bioimaging^23^, taste masking^19^, and even cell seeding scaffolds^20^.

Taking advantage of the physicochemical robustness of sporopollenin, several exine extraction methods using high temperature and corrosive chemicals have been established to prepare SECs^21^,^24^,^25^,^26^,^27^, all the while maintaining natural morphological characteristics including the intricate surface topography^9^,^25^. However, current efforts have been focused on pollen or spores from only a small subset of plant species including *Lycopodium clavatum*^3^,^15^,^17^,^19^,^22^,^23^,^24^ and *Helianthus annus*^16^. Indeed, SEC process development has been limited to micrometer thick-walled pollen species with high mechanical stability which facilitates successful preservation of microarchitecture features throughout the harsh processing steps. On the other hand, SEC development from submicron thin-walled pollen species remains elusive due to weaker mechanical properties which hinder faithful preservation of structural features. Considering that thin-walled pollen grains have greater aerosol dispersion^28^ and different mechanisms of digestion^29^ than thick-walled pollen grains, there is strong motivation for developing SECs from thin-walled pollen species towards realizing new applications such as aerosol drug delivery and taking advantage of SECs with different degradation behaviors.

One promising example of submicron thin-walled pollen species is *Zea mays* pollen, which presents attractive material properties originating from its status as the most common crop cultivated worldwide and its unique structural characteristics. *Zea mays* pollen grains boast a larger size in comparison to other species, ranging from 70 to 100 μm in diameter depending on the hydration conditions^30^. The multiple-layered coating of *Zea mays* pollen, consisting of an exine, intine, and a lipid fraction^31^,^32^, is thin (~1 μm)^33^ and elastic, yet robust enough to support its spherical shape without rupture^12^. To date, successful extraction of SECs from *Zea mays* pollen grains remains elusive. The first attempt to extract *Zea mays* sporopollenin was reported by Dominguez *et al.* while evaluating various pollen grains including those from *Betula alba*, *Ambrosia elatior, Zea mays* and *Pinus pinaster*^25^. Upon exhaustive chemical processing, all types of pollen particles demonstrated the absence of cellulose with negligible effects on the basic structure of the sporopollenin. However, morphological characterization of the end product showed crumbled structureless remains of *Zea mays* while all other species maintained their natural shape^25^. Although pollen harmomegathy (the natural folding or buckling behavior of pollen grains in response to desiccation) has been previously established and widely recognized as a natural defense mechanism against dehydration^7^,^8^,^11^,^34^, complete collapse of the *Zea mays* SECs was irrecoverable at the final stage, indicating that improvements to the processing protocol would be advantageous in order to produce technologically viable SECs.

Taking these factors into consideration, we have developed an improved method to obtain morphologically intact, thin-walled SECs from *Zea mays* pollen capsules using a one-pot acid hydrolysis process while bypassing complete dehydration in the intermediate stage. To our knowledge, this is the first report of thin-walled SECs with conserved inner structural features in both the microscale and nanoscale regimes. Scanning electron microscopy (SEM), dynamic image particle analysis (DIPA), CHN elemental analysis, and confocal laser scanning microscopy (CLSM) were employed for capsule characterization. SEM enabled high resolution imaging to accurately compare morphological characteristics of resulting SECs obtained through different processing strategies, and DIPA was employed for high-throughput size measurements and secondary morphological characterization. CSLM was also utilized for SEC morphological characterization. In addition, CHN elemental analysis provided compositional information relating to the degree of residual protein. Taken together, the findings indicate that thin-walled SECs can be obtained with 82% protein removal efficacy by using an acid hydrolysis extraction method in 85 (w/v)% phosphoric acid (H_3_PO_4_) within 2.5 hours.

## Experimental Section

### *Zea mays* Pollen Material

Defatted *Zea mays* pollen grains were obtained from GREER^®^ Source Materials (USA) and the as-supplied pollen were pretreated with ACS grade ethyl ether in order to remove the waxy coating and any other fatty acid constituents of the pollen grains.

### Extraction of *Zea mays* Sporopollenin Exine Capsules (SECs)

Three representative approaches to SEC extraction, including 6 M HCl hydrolysis, H_3_PO_4_ hydrolysis, and ethanol ultrasonication, were explored. (*1*) Hydrolysis with 6 M HCl; Two grams of defatted *Zea mays* pollen grains were suspended in 6 M HCl in a perfluoroaloxy (PFA) round bottom flask fitted with a glass condenser. Under gentle agitation, the solution was refluxed at 70 °C for 10 hours. The resulting SECs were collected with a vacuum filter and then washed with a series of organic and aqueous solvents as follows: 5× water (100 ml), 2× acetone (100 ml), 1 × 2 M HCl (100 ml), 5× water (100 ml), 2× acetone (100 ml), 1× ethanol (100 ml). In between each wash, SECs were left in suspension for at least five minutes then vacuum-filtered for solvent exchange. Upon completion of the solvent exchange serial washing, SECs were collected and dried at 60 °C for eight hours and stored for further characterization. (*2*) Acid hydrolysis with H_3_PO_4_; Treatment of *Zea mays* pollen is identical to the above protocol but with a different acid for the hydrolysis. (*3*) Ethanol ultrasonication; Two grams of defatted *Zea mays* pollen grains were suspended in 50 ml of ethanol and placed in bath sonication for one hour. After 5 mins of centrifuge at 3000 RPM, the supernatant was removed and replaced with fresh ethanol and then placed in bath sonication for an extra 30 mins. The ethanol was replaced again by a centrifugation step and washed with 25 ml of water, then placed in 50 ml acetone for another 30 minutes of bath sonication. After removing the acetone, the sample was washed with 25 ml of water five times, filtered, and dried at 60 °C for eight hours.

As for the new protocol to obtain intact *Zea mays* SECs, modification to the post-extraction processes was made as follows. The vacuum filtered SECs after acid hydrolysis are rinsed with fresh Milli-Q-treated water over a 20 cm × 20 cm precut nylon mesh (ϕ: 30 μm) (Elko Filtering, USA) placed within a clean beaker. The SEC suspension is kept submerged in water with gentle agitation for 5 mins, and then the mesh is transferred and soaked into a new beaker with fresh water preheated to 50 °C for 5 mins. The same strain-dialysis washing method is repeated with 50 °C Milli-Q water (4X), 50 °C acetone (2X), 50 °C 2M HCl (1X), 50 °C water (until neutralized at pH 7–8), 50 °C acetone (2X), then 50 °C ethanol (2X). After the final wash, the mesh is transferred into fresh water which is then heated to 80 °C for 30 mins under the fume hood to rid of all residual acetone and ethanol. The resulting SECs are removed from the mesh and stored in a 50 mL tube with 20 mL of Milli-Q water at 4 °C for further characterization.

### Elemental Analysis

Compositional analysis of *Zea mays* pollen grains and extracted SECs were conducted with a calibrated VarioEL III CHN elemental analyzer (Elementar, Germany). Approximately 5 mg of each dried sample were placed in an oven at 60 °C for at least one hour prior to CHN reading. For wet samples, 5 mL of the suspension were dried in the oven at 60 °C overnight prior to analysis. The protein concentrations for samples were calculated using the detected Nitrogen concentration and the Total Kjeldahl Nitrogen (TKN) conversion factor of 6.25 in accordance to recommendations from the Association of Analytical Communities (AOAC)^35^ as shown below:


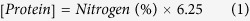


### Scanning Electron Microscopy (SEM)

SEM morphological characterization was performed on unprocessed pollen grains and processed SECs by employing an FESEM 7600F instrument (JEOL, Japan). Processed and unprocessed samples were coated with platinum at a thickness of 10 nm with JFC-1600 (JEOL, Japan) (20 mA, 60 sec). Samples were observed using an acceleration potential of 5.00 kV at different magnifications. In order to capture cross-sectional images of the samples, pollen grains were subjected to cryogenic freezing in liquid nitrogen. The fixated samples were then diced with a surgical blade and coated with platinum for observation.

### Dynamic Image Particle Analysis (DIPA)

The FlowCam^®^ instrument (Fluid Imaging Technologies, USA) was equipped with a 200 μm flow cell (FC-200) and a 20 X magnification lens (Olympus, Japan) and controlled by the VisualSpreadSheet version 3.4.11 software package. For sample preparation, 5 mg from each dry sample are suspended in 5 mL of water prior to measurements, whereas 1 mL from each wet sample is diluted with 4 mL of water for measurements. Measurements were collected automatically at a fixed rate of 10 frames s^−1^ and image collection was performed with an internal filter for particles ranging between 55–75 μm. A minimum of 100,000 particles was collected for each measurement and 1,000 highly focused particles from each image batch were selected by the edge gradient. Micromeritic analysis of particle morphology was evaluated based on diameter, aspect ratio, and circularity by circle-fit. The aspect ratio measures the length to width ratio of each particle while the circle-fit parameter numerates the deviation of the particle edge from an approximated best-fit circle assigned by the software.

### Confocal Laser Scanning Microscopy (CLSM)

Autofluorescence from pollen grains and SECs were observed with confocal laser scanning microscopy (Carl Zeiss LSM700, Germany) equipped with three spectral reflected/fluorescence detection channels, six laser lines (405/458/488/514/543/633 nm), and a Z1 inverted microscope (Carl Zeiss, Germany). Samples were fixated between two sticky slides (Ibidi, Germany) with a drop of Vecatashield^®^ Mounting Medium (Vector Laboratories, USA) and images were captured under an EC Plan-Neofluar 40×/1.30 Oil DIC M27 lens (Carl Zeiss, Germany) with the following settings: laser excitation wavelengths at 405 nm (10.0%), 488 nm (11.0%), and 561 nm (10.0%) detected with emission fitters 410–516, 493–556, and 566–685 nm, respectively; Under plane scanning mode, the laser scan speed was fixed at 67 sec per each phase and the pixel dwell timing was set at 12.6 μsec. All captured images were processed using ZEN 2008 software (ZEISS, Germany).

## Results and Discussion

**Figure 1:**
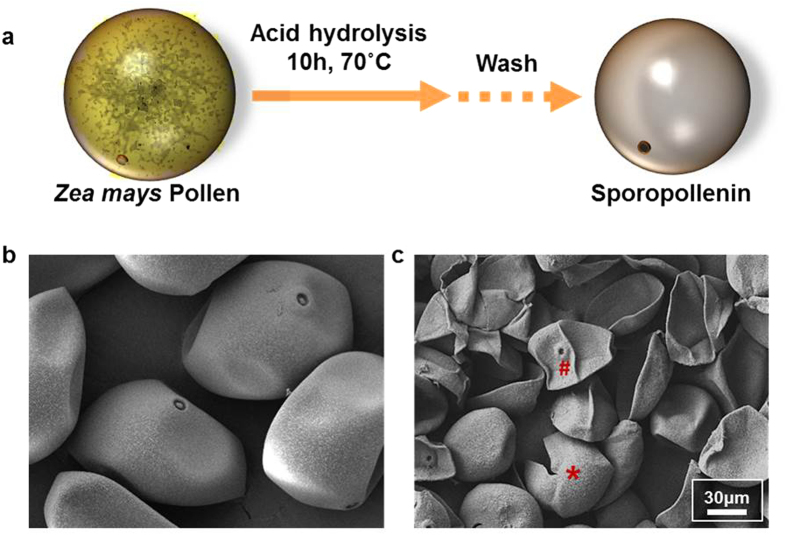
*Zea mays* sporopollenin exine capsule production scheme. **(a)** Cartoon schematic of conventional protocol consisting of an acid hydrolysis step followed by an extensive wash which is expected to produce capsules retaining the native form. SEM results obtained from conventional protocol show unprocessed pollen **(b)** and processed SECs **(c)** with severe rupturing (*) and buckling (#) after processing.

A key goal of this work is to develop a method whereby acid hydrolysis of *Zea mays* pollen grains can yield transparent spherical capsules completely devoid of sporoplasmic contents, in turn yielding protein-free capsules (Fig. 1a). To achieve this goal, *Zea mays* SEC extraction was initially performed using a one-pot processing method that followed a previously published protocol^36^,^37^. It consisted of a 85 (w/v)% H_3_PO_4_ reflux at 70 °C for 10 hours followed by a series of washing steps. The obtained capsules were then observed by SEM imaging along with unprocessed pollen grains for morphological comparison. The SEM images revealed that the initial pollen grains in their native form were not fully spherical. Instead, they appeared crinkled and lacked regular patterns or structures. Random buckling and depression along the grain surface was apparent, albeit the gross morphological features were intact (Fig. 1b). On the other hand, the processed capsules were all severely collapsed or cracked as seen in Fig. 1c, mirroring the findings of Dominguez *et al.*^25^.

**Figure 2:**
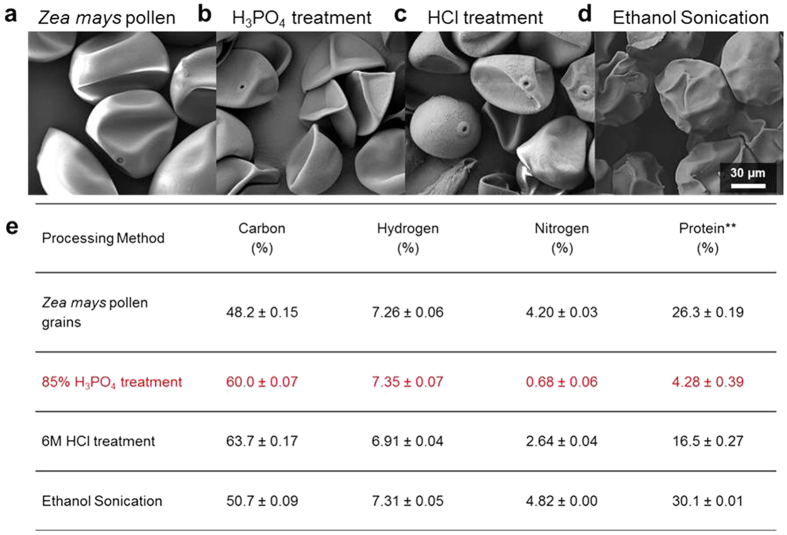
Comparison of *Zea mays* SECs extracted by varying methods observed under SEM. **(a)** Unprocessed *Zea mays* pollen after dehydration, **(b)** SECs from 10 h of acid hydrolysis in 85% H_3_PO_4_, **(c)** SECs from 10 h of HCl hydrolysis, and **(d)** SECs obtained from ethanol with sonication. **(e)** Chemical composition is analyzed by CHN analysis and reported as average values with standard deviation (n = 3). **Protein content is calculated with the Specific Jones Factor as outlined by the Kjeldahl method^33^.

Taking into account the negative effect of H_3_PO_4_ on the capsule morphology and the harsh treatment of SECs with acid hydrolysis^10^, two additional SEC extraction methods were introduced. In one method, SECs were extracted by the same acid hydrolysis method, but with HCl instead of H_3_PO_4_, which was chosen based on its cellulose degradability^38^. Additionally, SECs were prepared by an ethanol sonication method that has been previously suggested for delicate pollen grains^26^. In this second method, pollen grains were suspended in ethanol and were sonicated twice with replenishment of fresh ethanol in between the sonication rounds, then washed before further characterization. Morphological characteristics of the resulting SECs were analyzed with SEM and CHN elemental analysis was performed (Fig. 2). SEM images of the SECs prepared by the two acid hydrolysis methods using H_3_PO_4_ and HCl both showed buckling and general structural instability. A large fraction of the capsules was observed to be damaged from processing with all acid types. At higher magnification images, it was revealed that surface debris adhered to the SEC surface after HCl hydrolysis despite extensive washing, whereas the H_3_PO_4_-treated SECs showed minimal debris (Fig. S1). CHN results confirmed the superiority of H_3_PO_4_ treatment, recording 0.68% nitrogen content in the SECs compared to 2.64% nitrogen content for HCl-treated SECs.

By contrast, the SECs extracted from ethanol-sonication treatment revealed a strikingly unique appearance compared to the acid-hydrolyzed SECs. Most notably, the SECs from the ethanol-sonication treatment retained their spherical structures with little breakage, yet with a highly distorted surface topography that lacked the smoothness of unprocessed pollen. A condensed core structure was apparent within the SECs along with only limited buckling. However, CHN analysis showed no appreciable change in the chemical composition, indicating low protein removal efficacy. While the ethanol-sonication method is insufficient to reduce protein contents, it also provided evidence that an internal support structure could help to reduce the extent of capsule breakage. From these experiments, we conclude that acid hydrolysis with H_3_PO_4_ is the most suitable method to further explore on the basis of producing protein-free capsules.

**Figure 3:**
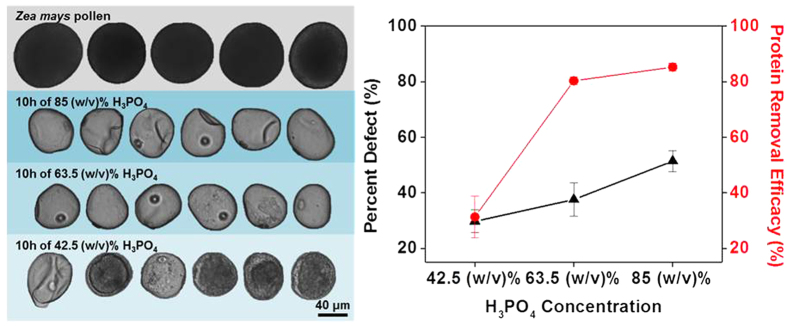
Comparative analysis of SECs obtained from treatment with different H_3_PO_4_ concentrations. The tested H_3_PO_4_ concentrations were 85 (w/v)%, 63.5 (w/v)%, and 42.5 (w/v)%. Morphological characterization by Dynamic Image Particle Analysis (DIPA) (scale bar = 40 μm) (left). Protein removal efficacy calculated from CHN analysis and percent defect calculated from particle tracking are expressed by the average and standard deviation (n = 3).

Motivated by the potential to structurally reinforce the capsules by filling in the empty cavity, we hypothesized that preserving a liquid environment inside the capsule would be sufficient to maintain the spherical morphology. Briefly, *Zea mays* SECs were extracted by the same 10 hour reflux in 85 (w/v)% H_3_PO_4_ at 70 °C, but washing was performed by dialysis in order to minimize the amount of time in air or under vacuum. As a result, the empty capsules would always be in a bloated state (see methods for a detailed description). After the final washing step with ethanol, the SECs were dialyzed with deionized water and kept suspended in water for storage. To avoid the dehydration requirement, DIPA analysis was implemented in place of SEM for comparative analysis of the two types of SECs obtained from the original protocol and the modified protocol. Comparative results indicate that the spherical morphology was largely preserved for most particles, albeit with some damaged particles remaining (Fig. S2). To improve the yield of morphologically preserved particles, SEC extractions with reduced H_3_PO_4_ acid concentrations of 63.5 (w/v)% or 42.5 (w/v)% were also tested. The extracted SECs were kept hydrated in order to maintain the spherical structure and the final products were evaluated with DIPA and CHN analysis as shown in Fig. 3. The SECs obtained from 63.5 (w/v)% H_3_PO_4_ showed promising results comparable to the quality of SECs obtained from 85 (w/v)% H_3_PO_4_ with calculated protein removal efficiencies of 80.3% and 85.2%, respectively, while improving the percent defect from 51.4% to 37.5%. SECs acquired from 42.5 (w/v)% H_3_PO_4_ treatment further reduced the prevalence of defective SECs down to 29.7% but this condition was insufficient for effective protein removal.

Interestingly, several intact SECs from the batch of 42.5 (w/v)% H_3_PO_4_ hydrolysis were seen with “yolk”-like structures with dense-colored boundaries (approximately 65% of the entire batch) either attached to the outer layer or loosely floating within the cavity in which the opaque traces were concentrated (Fig. S3). Similar artifacts were also observed in a small population of damaged particles remaining partially attached to the disfigured exine (Fig. S3). Based on the close resemblance in shading to unprocessed pollen and based on CHN analysis suggesting an approximate 69% retention of the original protein content, the core appeared to be a concentrate of the remaining sporoplasm while the dense boundaries were speculated to be the intine protecting the core. According to Zhang *et al.*, complete dissolution of cellulose in H_3_PO_4_ is achieved only when the acid concentration exceeds 81.7 (w/v)% whereas reversible swelling is expected at concentrations below 80 (w/v)%^39^. This trend agrees well with our observation of intact intine layers in the 42.5 (w/v)% H_3_PO_4_-processed SECs, whereas there was complete dissolution at higher acid concentrations.

**Figure 4:**
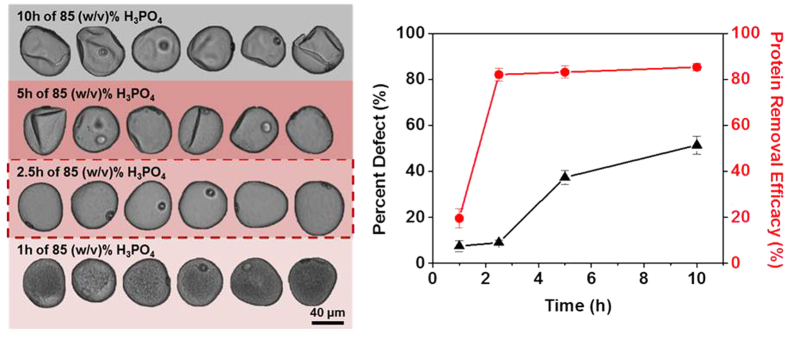
DIPA Images of SECs extracted by 85 (w/v)% H_3_PO_4_ at varying time points. Protein removal efficacy obtained from CHN analysis and percent defect measurement from particle tracking are expressed by the average and standard deviation (n = 3). The highlighted section on the graph corresponds to the dotted SEC treatment protocol which is determined to be the optimal condition for *Zea mays* SEC preparation.

Recognizing the need to remove the intine, the general mechanism of SEC extraction was treated as a two-step process: first, complete dissolution of the intine was treated as the rate-limiting step (Fig. S4), followed by removal of the remainder of sporoplasm in order to obtain an isolated exine capsule. To meet the needs for both steps, 85 (w/v)% H_3_PO_4_ was determined to be the most suitable option despite its corrosiveness. To improve the processing outcome, we next varied the duration of exposure time to the acid hydrolysis step. SECs were prepared with 85 (w/v)% H_3_PO_4_ in 70 °C and SEC samples were collected after 1, 2.5, and 5 hours incubation for DIPA and CHN analysis along with 10 hour processed SECs (Fig. 4). A progressive increase in percent defect was observed with extended exposure time, with a sharp increase, especially between 2.5 and 5 hours, from 9.0% to 37.3%. Meanwhile, the protein removal efficacy of H_3_PO_4_ appeared to reach its peak between 1 to 2.5 hours with an increased removal of total protein rising from 19.5% to 82.1%. Notably, the 1 hour-treated SECs exhibited a similar opaqueness to the 42.5 (w/v)% H_3_PO_4_-processed SECs, except the former also featured an intact intine ring (Figs 3 and 4). Putting the observations in perspective, the rate-limiting intine removal step required approximately one hour as evidenced by the absence of intine in the DIPA images and the sudden improvement in protein removal efficacy after the first hour. The next 1.5 hours appeared to be sufficient for removing the sporoplasm bulk as indicated by the degree of protein removal efficacy. Furthermore, the sudden increase in the defective capsules appeared to coincide with the depletion of the bulk content, which again emphasizes the importance of a structural support to maintain the intact spherical morphology of *Zea mays*. Collectively, the data support that 2.5 hour treatment in 85 (w/v)% H_3_PO_4_ is optimal among the tested conditions with only 9% defect and 82% protein removal efficacy.

**Figure 5:**
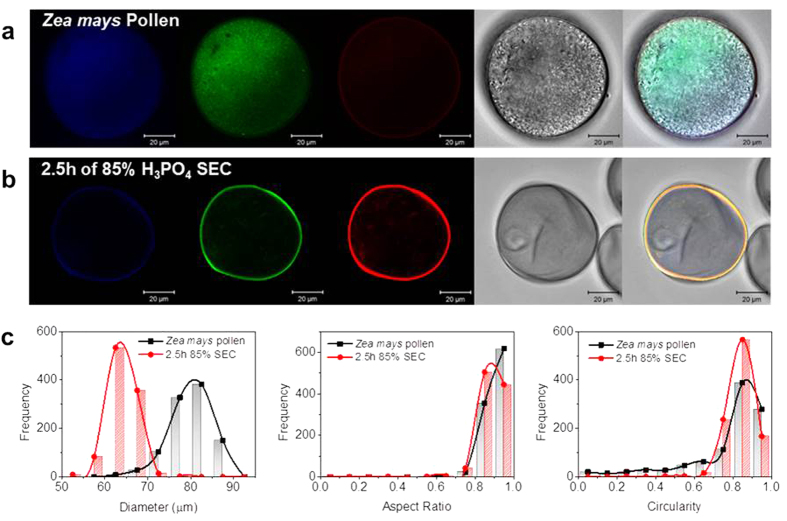
Quality evaluation of final SEC product conducted by CLSM and DIPA. **(a)** CLSM detected autofluorescence of unprocessed *Zea mays* pollen is presented for the blue, green, and red channels, respectively, and then merged with the corresponding DIC image. **(b)** Autofluorescence detection of SECs is also expressed in the same order for comparison. **(c)** Micromeritics of both unprocessed pollen and SECs analyzed in three categories for morphology: diameter, aspect ratio, and circularity.

In order to further characterize this optimal SEC preparation, CSLM imaging was conducted and detected the change in autofluorescence intensity in the pollen residues. Natural pollen particles have been previously reported to emit autofluorescence with wavelengths that are organelle-specific according to Pöhlker *et al.*^40^. The processed SECs and unprocessed pollen grains were observed under CLSM in order to compare the change in composition, as registered by differences in the autofluorescence. In the CLSM images of the unprocessed *Zea mays* pollen, a bright fluorescence was observed in the blue and green channels throughout the pollen cavity with a slight appearance in the red channel as well (Fig. 5a). When all fluorescence channels were merged on top of the differential interference contrast (DIC) microscope image, a homogenous blue-green fluorescent pollen particle was visualized with relatively low fluorescence around the borders. On the contrary, the CLSM image of the optimized SEC presented an intense fluorescence in the green and red channels while the blue channel was faintly visible (Fig. 5b). The merged image of the channels resulted in a bright yellow or orange fluorescence localized specifically on the pollen wall. Collectively, these results indicate successful clearance of sporoplasmic materials. Micromeritic analysis with DIPA measurements was also performed in order to evaluate morphological properties including diameter, aspect ratio, and circularity, respectively (Fig. 5c). As expected, a difference in diameter between the unprocessed grains and processed SECs was observed, with a significant ~20% size reduction from 80 μm to 64 μm on average. Meanwhile, both unprocessed pollen and SECs shared similar distribution profiles in aspect ratio and circularity concentrated at the high end of the spectrum indicating preservation of the original spherical conformation. In summary, SECs extracted by 85 (w/v)% H_3_PO_4_ for 2.5 hours were sufficiently free of sporoplasmic remnants and largely intact while retaining the characteristic structural appearance of the unprocessed grains. Moreover, the SECs were capable of being loaded with bovine serum albumin as a model compound and, importantly, SEM characterization indicated that drug loading provides structural support to preserve the inflated morphology of microcapsules even in the desiccated state (Fig. S5).

**Figure 6:**
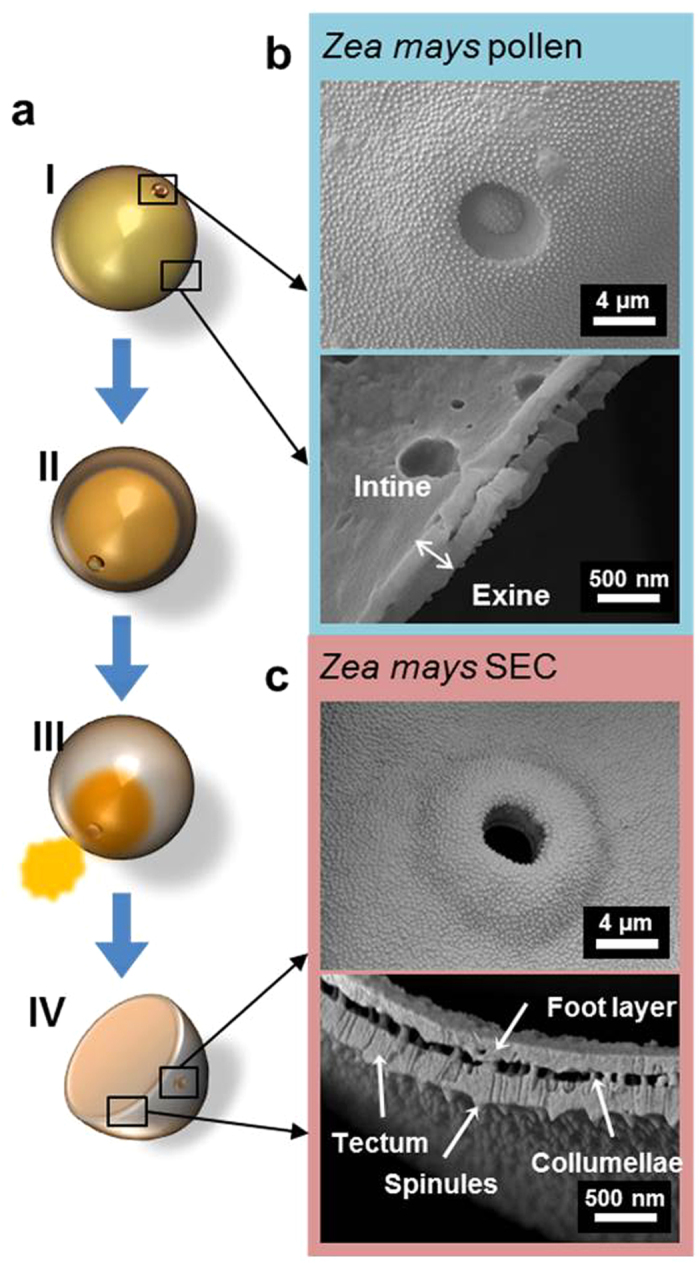
(**a**) Schematic illustration of SEC extraction process from pollen (left). Change in key features from the unprocessed pollen particle (I) to final SEC (IV) is observed by SEM. **(b)** Germination pore and sectional view of *Zea mays* pollen wall. **(c)** Germination pore and sectional view of *Zea mays* SEC wall is presented with labeled exine compartments.

In order to further characterize the unprocessed and processed samples, high-resolution SEM images of the SECs were compared to the unprocessed pollen grains, specifically focusing on key features of the pollen wall (Fig. 6). Following the scheme of the modified acid hydrolysis SEC extraction protocol, pre- and post-treated samples were collected, cryo-frozen, and fractured in order to obtain cross-sectional images of the pollen walls (Fig. 6a). The unprocessed pollen sample revealed a waxy overlay on the exterior and interior sides of the wall, presenting an overall smooth appearance (Figs 6b and S6). After having been exposed to H_3_PO_4_ treatment, the contents blocking the germination pore were removed, leaving behind the main aperture along with nanopores across the surface (Figs 6c and S6). The cross-sectional view of the ~500 nm-thick exine wall showed highly defined exine substructures including the outer tectum layer ornamented by spinules and the basal foot layer attached to the tectum by pillars of ~50 nm-diameter collumellae. Nanochannels were also seen throughout the tectum layer, giving it a streaky appearance (Fig. 6c). Taken together, the SEM micrographs demonstrate the effectiveness of the optimal H_3_PO_4_ treatment for removing exogenous debris from the wall surface while preserving all key structural features of the pollen wall.

**Figure 7:**
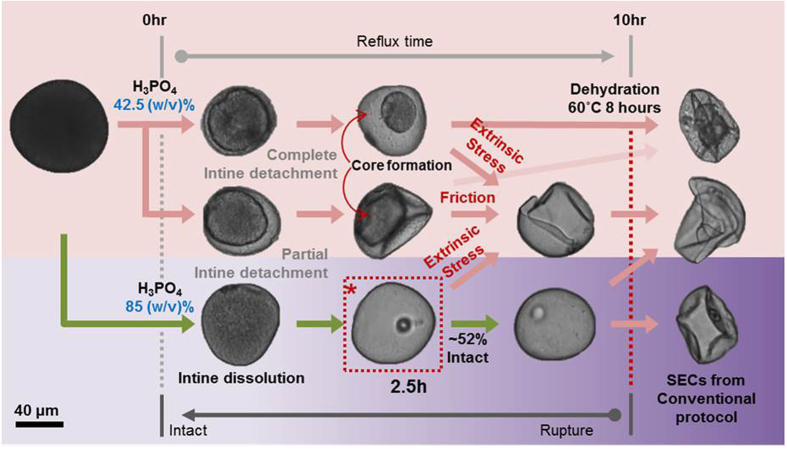
Mapping of the pollen-to-SEC transition showing different fates of SECs depending on processing conditions. Intine dissolution is the rate-limiting step to obtain SECs but the use of corrosive acid increases the chance of rupture with increasing time. Dehydration causes the flattening of capsules, and in some cases, causes rupturing of the capsules. The dotted box (*) is recommended as the optimal condition for *Zea mays* extraction.

Based on the results obtained in this study, a comprehensive outline of steps involved in extracting SECs from pollen grains is presented in Fig. 7. The rate-limiting step, which entails complete dissolution of the intine, is determined by the H_3_PO_4_ acid concentration. At acid concentrations below 80 (w/v)% H_3_PO_4_, the intine remains intact, shielding a large proportion of the sporoplasm from the acid resulting in SECs with high protein retention. When acid hydrolysis is conducted with 85 (w/v)% H_3_PO_4_, one hour is sufficient to achieve complete intine dissolution. The depletion of the sporoplasm follows soon after the removal of the intine, the bulk contents of the former are removed within 2.5 hours. Further refluxing up to ten hours, which was previously called for in the original protocol^36^,^37^, had negligible effect in improving the cleanliness of the SECs and adversely increased the proportion of defects. The overlap in the critical point of the increase in SEC breakage rates versus the plateau in protein removal efficacy (cf. Fig. 4) suggests that the bulk sporoplasm content helped stabilize the SEC structure before its removal. The effect of BSA loading on preserving the microcapsule’s structural integrity upon desiccation further supports this notion. Upon losing this structural support, a progressive loss of intact SECs becomes unavoidable. Also, a complete collapse in morphology is accompanied by dehydration further contributing to the lack of structural support. Hence, 2.5 hour treatment with 85 (w/v)% H_3_PO_4_ is determined to be the optimal processing scheme for *Zea mays* SEC production based on the tested conditions. We also wish to comment on the protein removal efficacy. While ~85% protein was removed from the SECs, there was a small remaining fraction based on the CHN analysis that should be considered in the context of *in vivo* applications and potential risk of allergenicity. In principle, the residual protein could affect the quality of drug loading although the preliminary BSA encapsulation data obtained in this study supports that the drug loading in the thin-walled pollen microcapsules is feasible and, in fact, beneficial. Looking forward, the addition of a benign surfactant treatment step may be considered among the possibilities to fully remove protein depending on the application need.

## Conclusions

To date, SEC extraction from pollen grains has been limited to thick-walled, rigid pollen grains. The fundamental problem limiting the use of thin-walled pollen has been the lack of rigid structure in the exine wall and, in turn, reliance on the intine and sporoplasm for structural support. With the disintegration of its internal counterpart and corrosive effects of the treatment conditions, the exine sheath becomes susceptible to extrinsic forces and fragile from the chemical treatment eventually leading to structural failure. The required dehydration step in the conventional protocol further destabilizes the structure, resulting in complete morphological loss.

Through the systematic approaches taken here, various factors involved in acid hydrolysis of natural *Zea mays* pollen grains were evaluated. Based on our findings, we have outlined a two-step model whereby SECs are produced starting with intine removal followed by sporoplasm removal, and the protocol was optimized to maintain particle morphology. The required dehydration step was also avoided in order to bypass the natural tendency of pollen walls to buckle when exposed to drying and to address the need for structural support to maintain the original shape of *Zea mays* pollen. Taking all matters into consideration, a reconstructed protocol is proposed for the extraction of *Zea mays* SECs which consists of a 2.5 hour reflux in 85 (w/v)% H_3_PO_4_ followed by dialysis with a series of solvents for washing, and finally, storage in an aqueous suspension. Based on this approach, successful *Zea mays* SEC production is reported for the first time with an optimized SEC extraction protocol. This new protocol opens the door to investigating a wide range of previously unexplored pollen species, especially those with thin and fragile walls which are attractive candidates for aerosol drug delivery and possess more sensitive degradation behaviors that could be exploited for controlled release. Also, the identified effects of pollen folding during SEC production will be useful in significantly improving product quality regardless of the pollen species and can even be exploited for applications that require controlled cycling of dehydration-rehydration. Lastly, the prodigious and renewable abundance of *Zea mays* pollen worldwide brings great promise towards scaling up the methodologies reported in this work towards generating renewable supplies of natural microcapsules.

## Additional Information

**How to cite this article**: Park, J. H. *et al.* Inflated Sporopollenin Exine Capsules Obtained from Thin-Walled Pollen. *Sci. Rep.* **6**, 28017; doi: 10.1038/srep28017 (2016).

## Supplementary Material

Supplementary Information
