# Supplementary material for: Inflated Sporopollenin Exine Capsules Obtained from Thin-Walled Pollen
Source: Sci Rep. 2016 Jun 15;6:28017. doi: 10.1038/srep28017 (PMC4908411; doi:10.1038/srep28017)
Supplement: Supplementary Information [file srep28017-s1.pdf]

Inflated Sporopollenin Exine Capsules Obtained from Thin-Walled Pollen

Supporting Information

Jae Hyeon Park[†,‡], Jeongeun Seo[†,‡], Joshua A. Jackman[†,‡], Nam-Joon Cho[†,‡,§]

[†]School of Materials Science and Engineering Nanyang Technological University, 50 Nanyang Avenue 639798, Singapore
[‡]Centre for Biomimetic Sensor Science, Nanyang Technological University, 50 Nanyang Drive 637553, Singapore
[§]School of Chemical and Biomedical Engineering, Nanyang Technological University, 62 Nanyang Drive 637459, Singapore

E-mail: njcho@ntu.edu.sg

Supporting Information

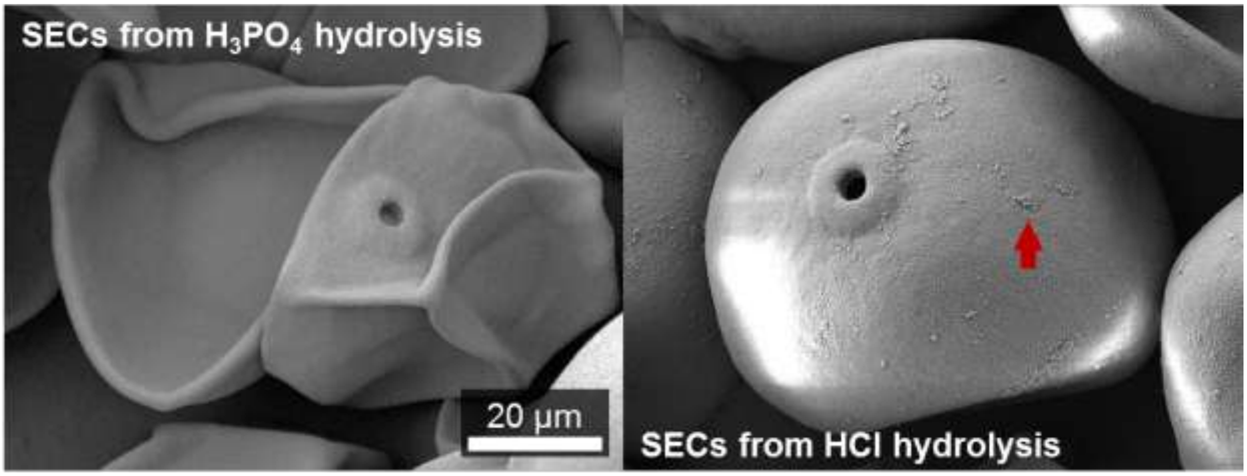

Figure S1. SEM images of surface debris (red arrow) observed on SECs extracted from H_3PO_4 (left) and HCl (right).

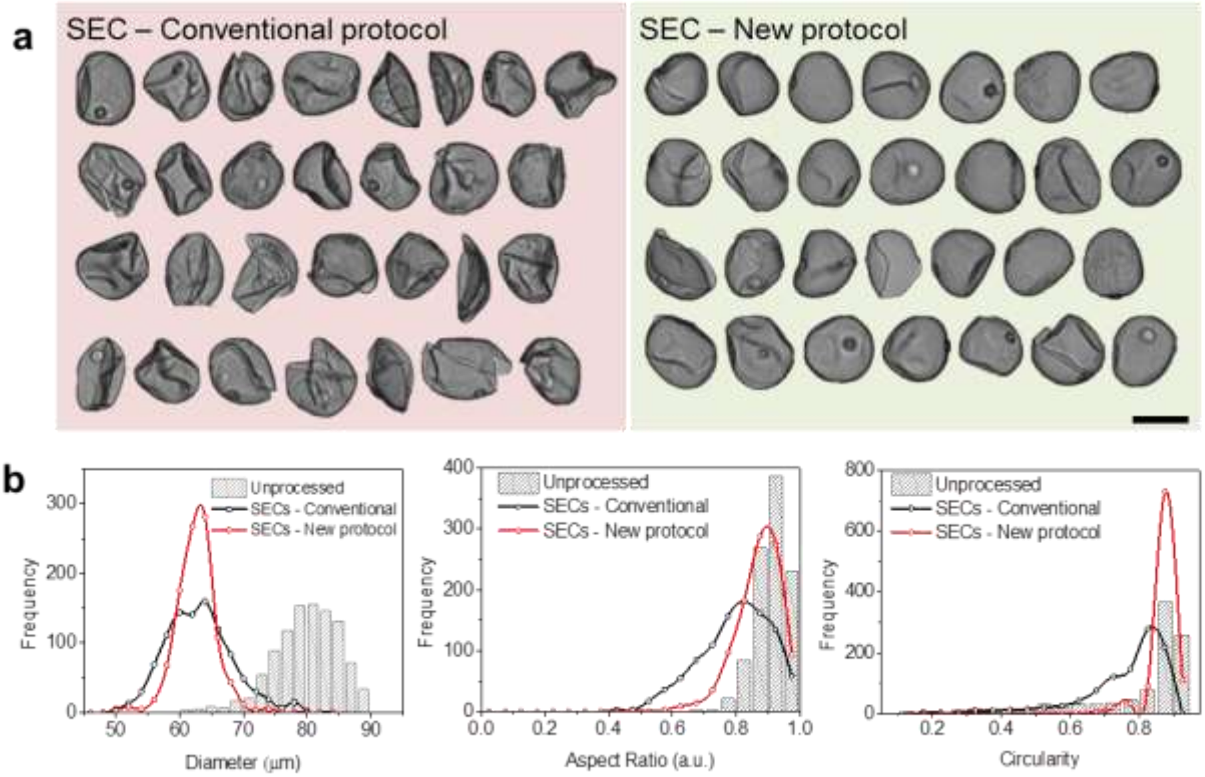

Figure S2. SEC images and analyses obtained from DIPA. (a) Morphological comparison of the SECs from the conventional protocol and from the modified protocol. **(b)** Micromeritics of the two SECs are compared against the unprocessed pollen.

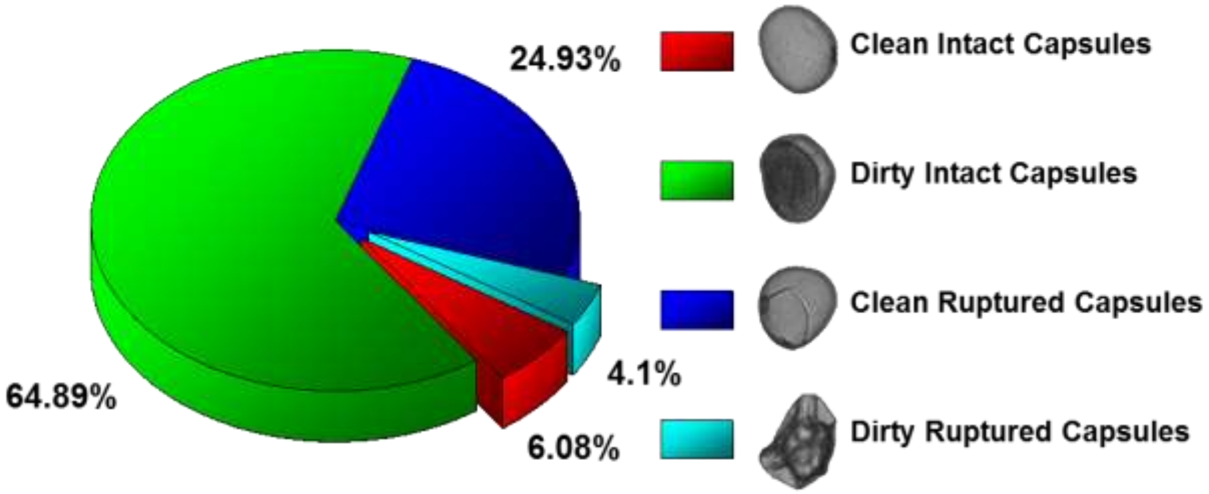

Figure S3. Evaluation of 1000 SECs collected from 10 hours of 42.5 (w/v)% H_3PO_4 hydrolysis. The collected SECs have been categorized into four groups according to their phenotype and the proportions of each group were calculated. A representative image from each category is displayed with the description of the phenotypes.

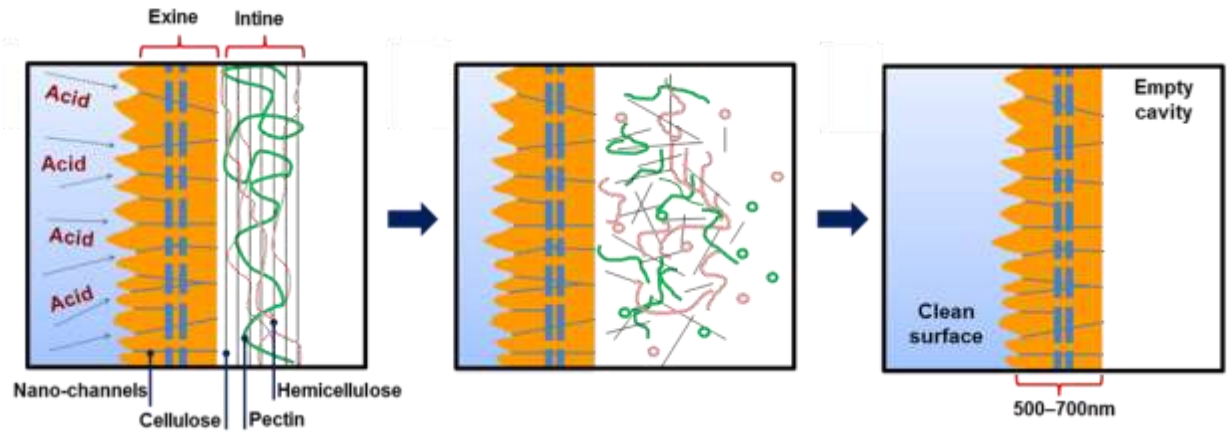

Figure S4. The acid penetrates through the nano-channels of the exine layer to breach the intine structure but must be sufficiently concentrated to disrupt the cellulose structure. The cellulosic materials are segmented and eventually excreted out from the complex to achieve a pure SEC.

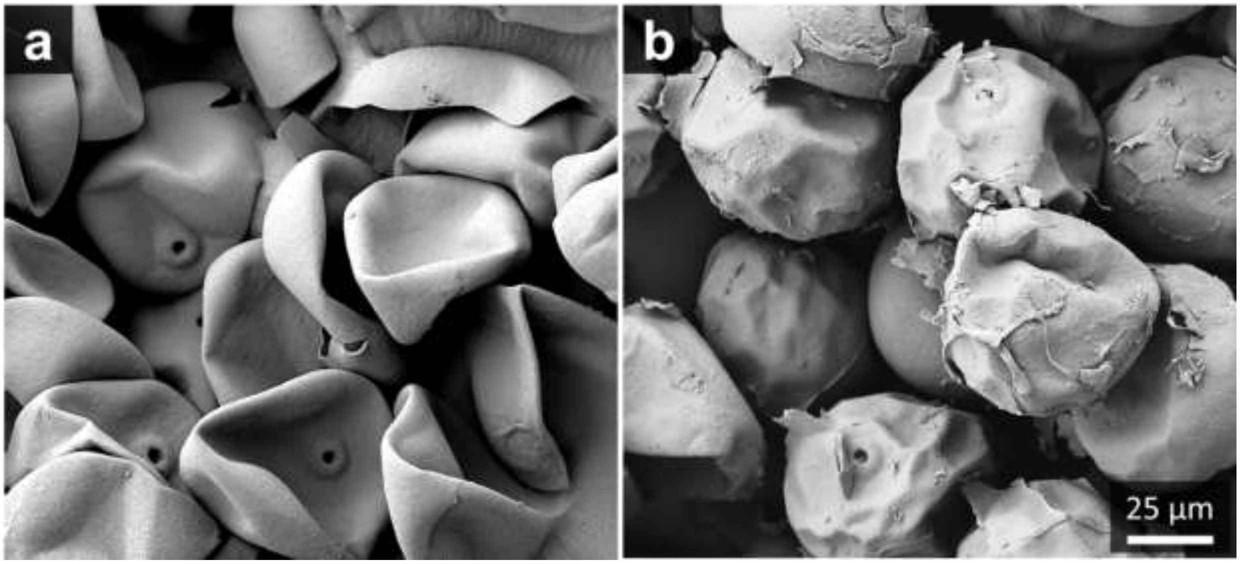

Figure S5. Loading of bovine serum albumin (BSA) into *Zea mays* SECs. Scanning electron microscopy images of dried Zea mays (a) before and (b) after encapsulation of 20 mg/mL bovine serum albumin (BSA). For protein loading, BSA is dissolved in a liquid suspension of *Zea mays* SECs, and then freeze-dried for 24 hours to allow passive encapsulation. The samples were dried for an extra hour at 60°C prior to SEM imaging. The successful loading of BSA into the SECs is denoted by the inflated morphology even in the desiccated state. Note that some residual protein remains on the surface, and this protein could be removed by more extensive cleaning studies although the focus here was on proof-of-concept loading so we focused on loading the maximum amount.

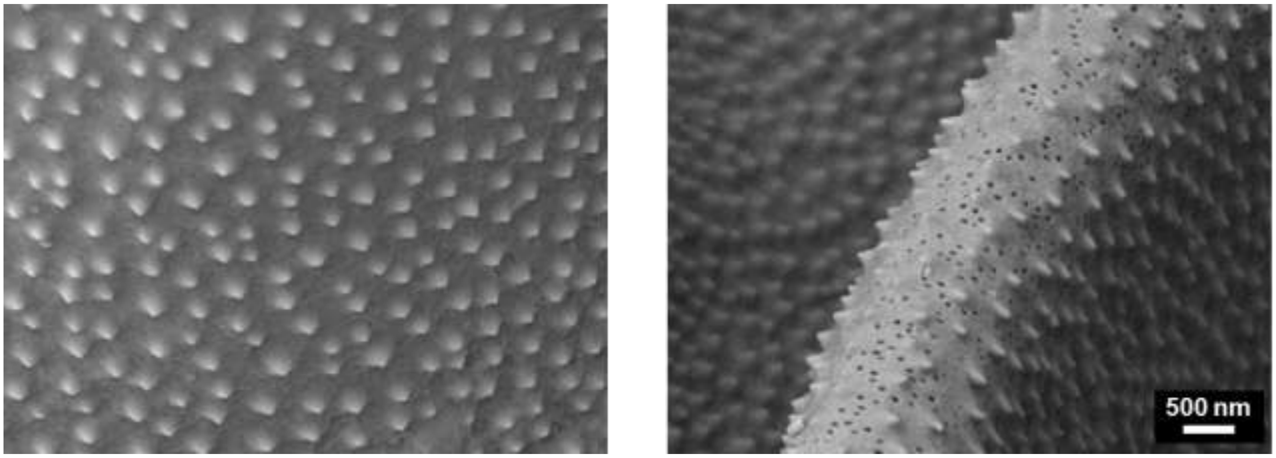

Figure S6. Surface morphology of the unprocessed pollen grain (left) and the SEC (right) observed under SEM to capture the topography of the nanoholes and spinules.
